# The ETS Transcription Factors ELK1 and GABPA Regulate Different Gene Networks to Control MCF10A Breast Epithelial Cell Migration

**DOI:** 10.1371/journal.pone.0049892

**Published:** 2012-12-20

**Authors:** Zaneta Odrowaz, Andrew D. Sharrocks

**Affiliations:** Faculty of Life Sciences, University of Manchester, Manchester, United Kingdom; National Cancer Institute, United States of America

## Abstract

Members of the ETS transcription factor family often target the same binding regions and hence have the potential to regulate the same genes and downstream biological processes. However, individual family members also preferentially bind to other genomic regions, thus providing the potential for controlling distinct transcriptional programmes and generating specific biological effects. The ETS transcription factor ELK1 controls cell migration in breast epithelial cells through targeting a cohort of genes, independently from another family member GABPA, and therefore achieves biological specificity. Here, we demonstrate that GABPA also controls cell migration in breast epithelial cells. However, GABPA controls the expression of a different network of target genes to ELK1. Both direct and indirect target genes for GABPA are identified and amongst the direct targets we confirm the importance of *RAC1* and *KIF20A* for cell migration. Therefore, although ELK1 and GABPA ultimately control the same biological process, they do so by regulating different cohorts of target genes associated with cytoskeletal functions and cell migration control.

## Introduction

Eukaryotic transcription factors are grouped into families based on their common DNA binding domains. Due to their similarity of their DNA binding domains, proteins within families have the potential to bind to similar DNA motifs and this has been shown to be the case for the ETS transcription factors where only subtle differences in binding specificity can be observed *in vitro* [1]. Given that there are 28 ETS family members in mammals (reviewed in [2]–[3]) and that they possess a similar binding potential it is unclear how biological specificity is achieved. However, insights into this have been provided by several genome-wide ChIP-seq/ChIP-chip studies, where it is clear that although there is substantial overlap in DNA binding *in vivo*, individual family members preferentially bind to subsets of sites. It seems likely that binding to these ‘exclusive’ sites accounts for the specificity of action of particular ETS factors ([4]–[7]; reviewed in [3]). Indeed, we recently showed that in breast epithelial MCF10A cells, ELK1 binds to DNA *in vivo* in two distinct manners, either overlapping with binding of another ETS protein GABPA (termed ‘redundant’) or binding to a different set of sites to GABPA (termed ‘unique’) [7]. Importantly, ELK1 was shown to control cell migration and it does so through regulating the expression of genes associated with ‘unique’ ELK1 binding sites. This study therefore confirmed the hypothesis that a specific biological effect can be elicited by the binding of a single family member, in this case ELK1, to a series of target genes that are not targeted by other family members.

In addition to the specific role for ELK1 in controlling MCF10A cell migration, a large number of genes targeted by ELK1 overlap with the binding of GABPA (ie the ‘redundant’ class [7]). Similarly, in human T cell lines, GABPA binding substantially overlaps that of the other ETS proteins ETS1 and ELF1 [4], [5]. In this overlapping binding mode, GABPA is thought to control the activities of housekeeping genes such as those encoding ribosomal proteins. However, it is not clear whether GABPA functions to control specific sets of genes in an independent manner from other ETS proteins and hence drive distinct biological processes. Such a specific function appears likely, as GABPA has previously been associated with controlling many different processes. For example, it was recently demonstrated to play an important role in haematopoietic stem cell maintenance and differentiation [8]. It also has a role as a controller of cell cycle progression [9] and is important for the formation of a functional postsynaptic apparatus in neurons [10]–[11]. These studies suggest that GABPA likely binds in a ‘unique’ manner to sets of genes controlling these processes.

In this study we investigated the functional role of GABPA in MCF10A cells. As our previous results showed that ELK1 controls breast epithelial cell migration and this happens through regulating a set of target genes that are apparently ‘unique’ to ELK1 and not also bound by GABPA [7], we therefore assumed that GABPA would not affect cell migration and instead would control different biological processes. However, further investigation demonstrated that depletion of GABPA also induces a migratory defect in breast epithelial cells, suggesting that GABPA also controls the expression of genes important for this process. We further investigated the role of GABPA in controlling cell migration and demonstrate that although ELK1 and GABPA ultimately control the same biological process, they do so by regulating largely distinct transcriptional programmes.

## Results

### GABPA controls cell migration

**Figure 1:**
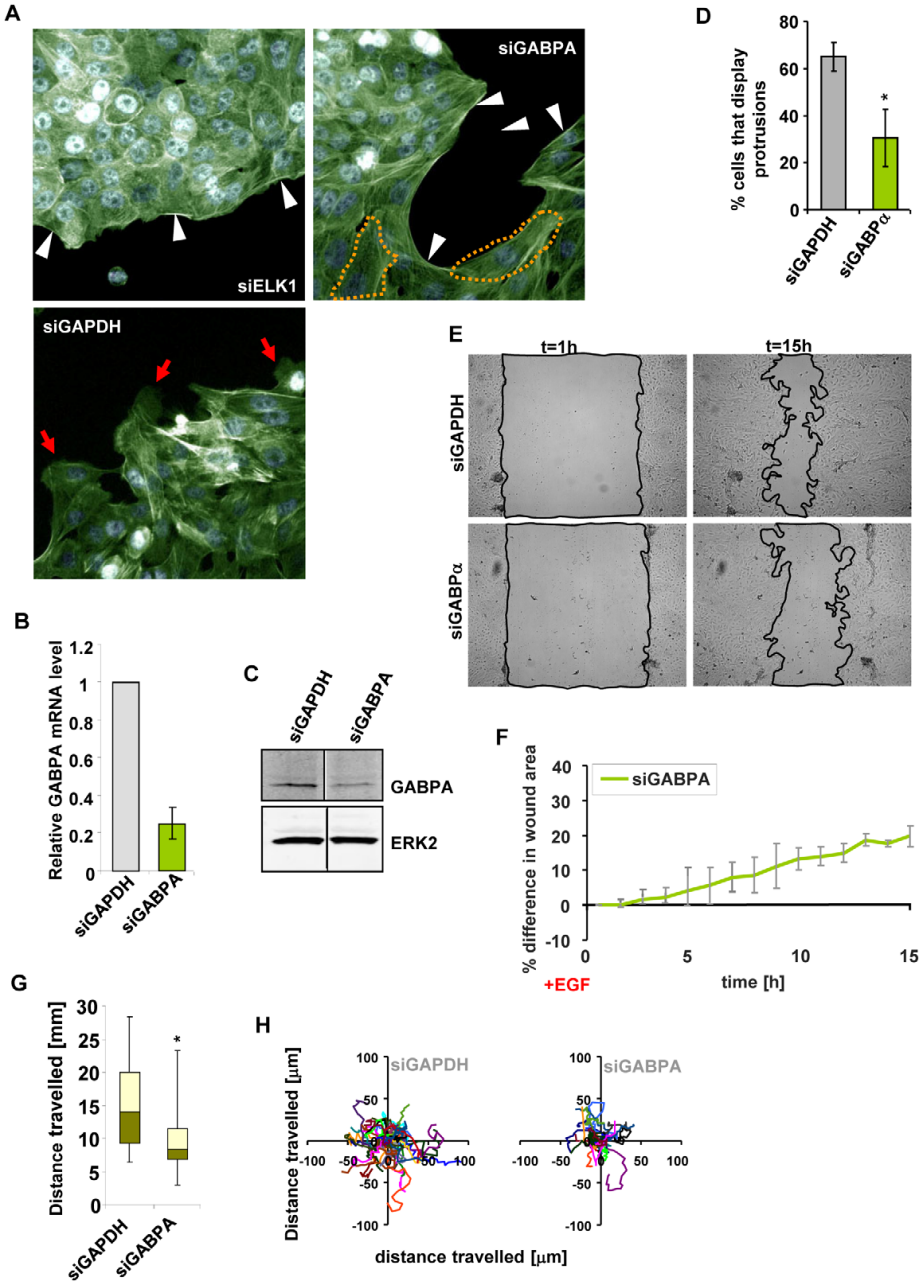
Depletion of GABPA affects the cytoskeleton and migratory properties of MCF10A cells. (**A**) Immunofluorescent images of MCF10A cells transfected with the indicated siRNA species, starved for EGF for 48 hours, stimulated with media containing EGF for 24 hours and stained with phalloidin and with Hoechst dye to visualise the actin cytoskeleton and nuclei, respectively. Red arrows – membrane protrusions, white arrowheads – subcortical actin, dashed lines – enlarged cell bodies. (**B**) RT-PCR quantification of the effect of siGABPA transfection on GABPA mRNA levels. Chart shows average values from three biological repeats with standard deviation. Statistical significance was determined in Student's t-test (*P<0.01). (**C**) Western blot analysis showing the effect of siGABPA transfection on GABPA mRNA levels. ERK2 levels are shown as a loading control. Each lane was taken from the same western blot to eliminate irrelevant lanes. (**D**) Quantification of the percentage of cells located on the edges of clusters exhibiting membrane protrusions upon transfection with siGAPDH (control) or siGABPA. Bars show average values from three biological repeats with standard deviations; in each repeat, three fields were scored. (**E**) Representative images of wounds created in monolayers of MCF10A cells transfected with the indicated siRNA species, starved for EGF for 48 hours, subsequently stimulated with media containing EGF and imaged for 15 hours. Black line marks borders of cell-free areas. (**F**) Cell-free areas in the images obtained as described in (E) for the siGABPA transfection were measured at hourly intervals and normalised to siGAPDH-transfected control. Shown are average values from three biological repeats with standard deviations. (**G and H**) MCF10A cells were transfected with the indicated siRNAs, starved for EGF for 48 hours, stimulated with media containing 20 ng/ml EGF and imaged for 24 hours. (**G**) Box plots show the distributions of lengths of trajectories travelled by MCF10A cells transfected with the indicated siRNA species between t = 1 h and t = 7 h of imaging. Data was obtained in four biological repeats of the experiment, and in each case ten cells were manually tracked. The green and pale yellow areas correspond to the second and third quartile of the distribution, respectively. The shaded area represents the distribution of distances covered in control siGAPDH-transfected cells. P-values were obtained in a Smirnov-Kolomogorov test (*P<0.05). (**H**) Distribution of the trajectories travelled by cells plotted in (G).

We previously demonstrated that depletion of the ETS transcription factor ELK1 in breast epithelial MCF10A cells leads to changes in the actin cytoskeleton, and in particular a loss of membrane protrusions and an accumulation of sub-cortical actin (Fig. 1A) [7]. This previous study indicated that this effect was largely driven by genes uniquely targeted by ELK1, independently from another ETS protein GABPA. Nevertheless, in a control experiment, we wanted to check whether GABPA might also have a role in the correct formation of the actin cytoskeleton in MCF10A cells, and so we depleted GABPA (Fig. 1B and C) and visualised the actin cytoskeleton by phalloidin staining (Fig. 1A). To our surprise, cells depleted of GABPA accumulated subcortical actin and often became enlarged. Moreover, while control siGAPDH-treated cells often exhibited membrane protrusions in response to EGF stimulation, as is characteristic of migratory cells, cells depleted of GABPA displayed fewer such protrusions (Fig. 1A and D). Given this latter observation, we also tested whether GABPA-depleted cells showed migratory defects. Wound healing assays demonstrated that GABPA-depleted MCF10A cells failed to properly respond to EGF treatment and wound closure was significantly delayed (Fig. 1E and F). This effect was specific as it could be reproduced with an alternative GABPA siRNA construct (Fig. S1). This result is suggestive of a migratory defect but could also be due at least partially to reduced proliferation. To more clearly demonstrate a defect in cell migration we used single cell tracking and, importantly, this also revealed defects in the migratory properties of MCF10A cells upon GABPA depletion (see Fig. 1G and H).

Together, these results demonstrate that GABPA plays an important role in controlling correct cytoskeletal formation which potentially links to a role in regulating the migration of MCF10A cells.

### The GABPA-dependent gene regulatory network

**Figure 2:**
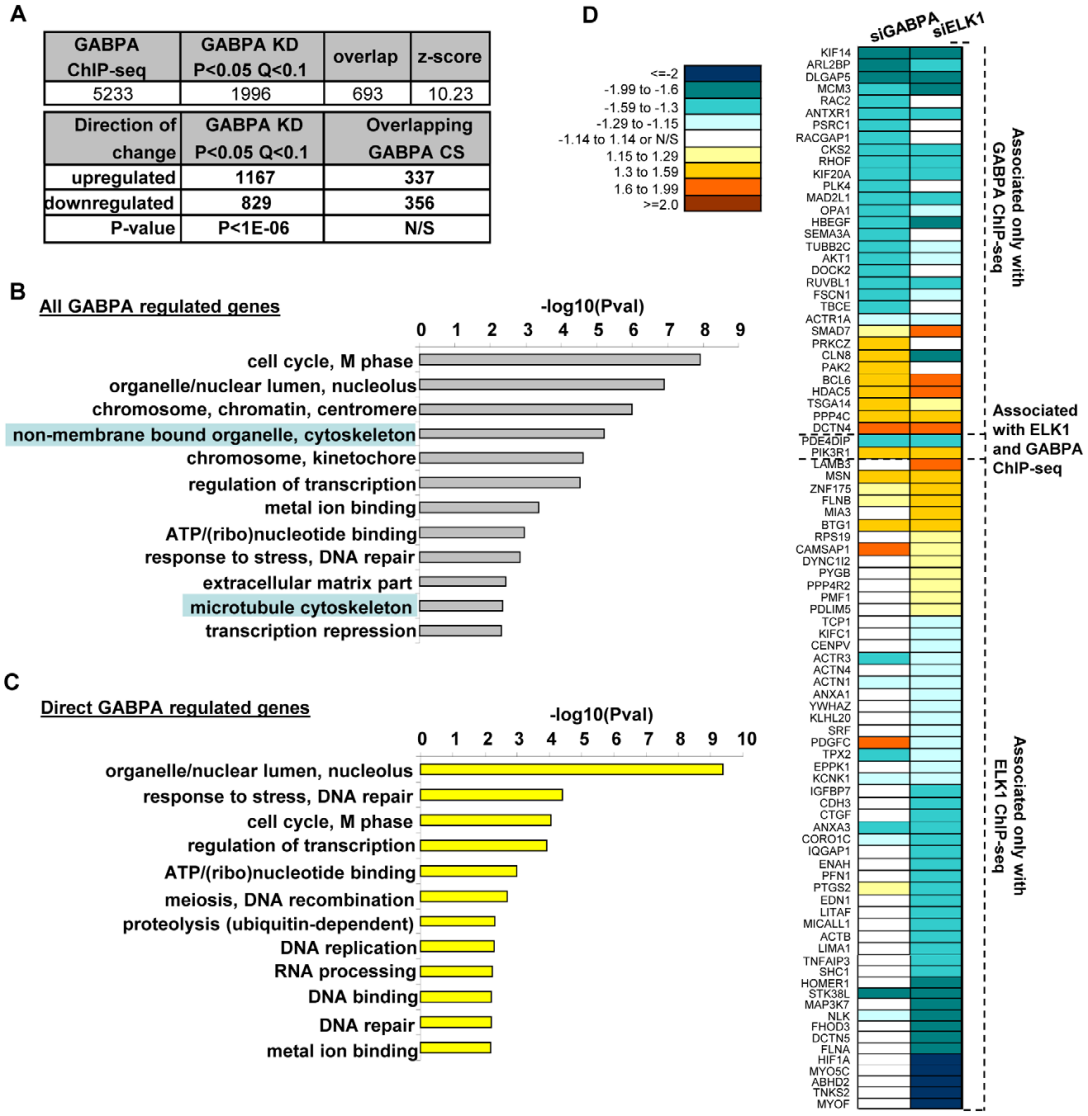
GABPA directly regulates gene expression in MCF10A cells. (**A**) Summary of the effect of siGABPA transfection on the transcriptome of MCF10A cells. Top – overlap of genes which change expression in cells depleted of GABPA (GABPA KD) with genes associated with GABPA binding regions as determined in a ChIP-seq experiment [12]. Bottom – distributions of up- and down-regulated genes in the whole dataset, or only for the overlap with ChIP-seq (GABPA CS) data. P-values were obtained in a chi square test. (**B and C**) Results of DAVID gene ontology analysis of lists of all genes showing a change of expression in MCF10A cells depleted of GABPA (**B**) or only those additionally associated with GABPA ChIP-seq regions (**C**). Bars indicate statistical significance of arbitrarily summarised clusters of terms (shown on the left). (**D**) Heatmap showing fold changes in mRNA levels of direct GABPA and ELK1 target genes (indicated on the right) coding for cytoskeleton-, migration- and adhesion-related proteins in MCF10A cells transfected with the indicated mRNA species, normalised to control (siGAPDH transfection).

The observation that GABPA plays a role in controlling cell migration was unexpected, as we previously showed that ELK1 controls this process in MCF10A cells, and it does this through a network of target genes in a manner that is independent of GABPA [7]. Therefore to provide an insight into how GABPA might be controlling cell migration, we depleted GABPA and used microarrays to examine the resultant changes in gene expression profiles in MCF10A cells. Overall, 1996 genes showed significant expression changes upon GABPA depletion, with most (58%) showing upregulation (Fig. 2A; Table S1). To determine whether the gene expression changes are likely directly or indirectly caused by GABPA, we took advantage of a published ChIP-seq dataset for GABPA in Jurkat cells [12]. This analysis revealed a highly significant overlap between GABPA binding and GABPA-dependent gene regulation, with a total of 693 (35%) of the deregulated genes corresponding to direct targets for GABPA, despite the different cell types analysed (Fig. 2A; Table S1). These direct targets were equally distributed between up- and downregulated genes, suggesting that GABPA might have both activating and repressive properties and that the bias towards upregulation observed for the whole transcriptome may be attributable to indirect effects. In contrast, little overlap was seen between the genes deregulated by GABPA loss and genes whose regulatory regions are bound by ELK1 (Fig. S2). Next, we used gene ontology (GO) analysis to assess the processes associated with the genes deregulated upon GABPA depletion. A number of functional categories were enriched, including several terms associated with the cell cycle, but also additional terms associated with the actin cytoskeleton (Fig. 2B). Further GO term analysis on the genes directly regulated by GABPA (i.e. both bound and deregulated) still returned terms associated with the cell cycle but those associated with the cytoskeleton were absent (Fig. 2C). This suggests that GABPA has a major direct role in cell cycle control as reported previously [9] but it mainly controls genes associated with the cytoskeleton in an indirect manner.

Although, the majority of regulation of cytoskeletal genes by GABPA appears to be indirect, we sought evidence that GABPA might also influence the formation of the actin cytoskeleton and cell migration in a more direct manner by acting through a more limited number of genes that are not abundant enough to constitute an over-represented GO term category. To test this, we manually extracted all the genes coding for cytoskeletal-, migration-, and adhesion-related proteins from the dataset of genes bound and regulated by GABPA, and looked at their expression in more detail (Fig. 2D). Of the 34 genes that matched this description, 70% showed downregulation upon GABPA depletion, indicating that GABPA acts predominantly as an activator in this context (Fig. 2D, top). Importantly, only two of these directly regulated genes were shown by ChIP-seq to be occupied and regulated by ELK1 in MCF10A cells (Fig. 2D) [7]. However, despite the lack of apparent ELK1 occupancy, a number of the genes directly regulated by GABPA were also deregulated upon ELK1 depletion, suggesting that an indirect mechanism is involved. Nevertheless, a number of these direct GABPA target genes are downregulated upon GABPA depletion but not following ELK1 depletion (eg *RAC2*, *RACGAP1*, *SEMA3A*; Fig. 2D) demonstrating the unique activity of GABPA in this context. Conversely, there are also a large number of genes associated with cytoskeletal and migratory functions that are bound and regulated by ELK1 and again ELK1 acts predominantly as a transcriptional activator in this context (Fig. 2D, bottom). Only a small proportion (27%) of these direct ELK1 target genes are also deregulated upon GABPA depletion, reinforcing the notion that ELK1 has a specific activity in directly regulating the expression of a large cohort of genes involved in these cellular functions.

Together these results demonstrate that GABPA controls the expression of a large number of genes associated with the formation of the actin cytoskeleton and required for cell migration. Comparisons with ELK1 reveal that there are a number of shared target genes but GABPA and ELK1 each control the expression of a group of specific target genes within these functional categories.

### GABPA controls an integrated network of cytoskeleton-related genes

**Figure 3:**
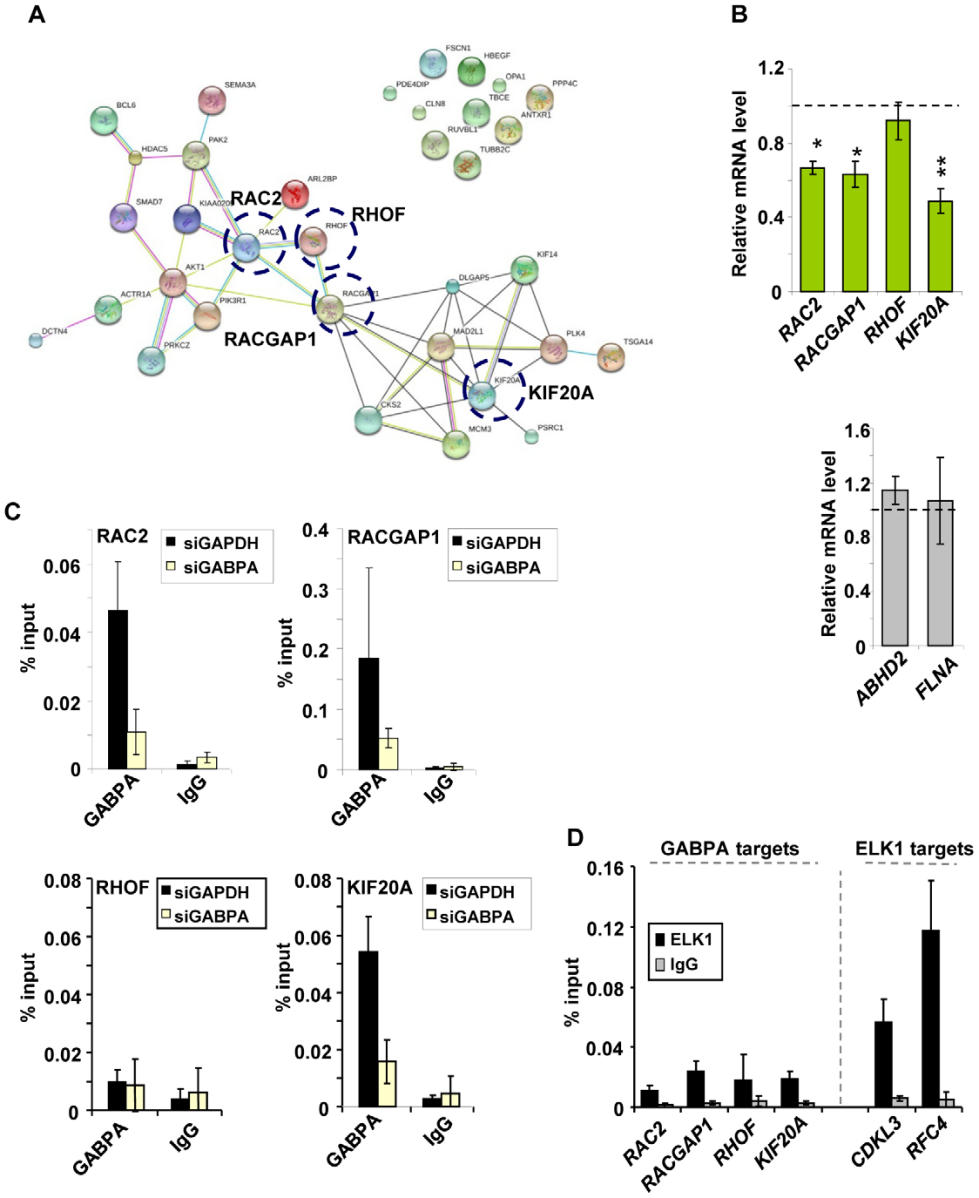
GABPA controls the expression of a network of cytoskeleton-related genes. (**A**) A STRING-derived network of proteins encoded by all genes that exhibit a statistically significant change of expression in MCF10A cells depleted of GABPA, that are associated with regions bound by GABPA, and that belong to GO terms associated with the cytoskeleton, cell migration or adhesion as determined by DAVID analysis. Proteins are circled whose encoding genes were chosen for further analysis. (**B**) The effect of siGABPA transfection on the expression of genes encoding proteins highlighted in panel A (green) and two negative controls (not GABPA targets; grey). Bars show average values from three biological repeats with standard deviation. Statistical significance was determined in paired Student's t-tests (*P<0.05, **P<0.01). (**C**) Charts show the binding levels of GABPA to DNA regions associated with genes encoding proteins highlighted in panel A, as determined in ChIP-qPCR experiments in MCF10A cells transfected with the indicated siRNA species and starved for EGF for 48 hours. IgG immunoprecipitation indicates the level of non-specific binding. (**D**) ChIP-qPCR of ELK1 occupancy on regions tested in (**C**) and on two positive control regions (associated with *CDKL3* and *RFC4*).

GO term enrichment suggested that GABPA, either directly or indirectly, controls the expression of groups of genes associated with the actin cytoskeleton and cell migration. Many of the changes in gene expression that occur upon GABPA depletion are moderate, despite the strong phenotype we see, and part of the reason for this could be that the GABPA target genes might be functionally interconnected. To test this, we used STRING [13] to investigate whether GABPA-regulated genes in these functional categories formed networks. Extensive interconnectivities were seen between GABPA targets within this network (Fig. S3) thereby providing a molecular rationale for how GABPA might control the formation of the actin cytoskeleton and cell migration. Such interconnectivities are not necessarily expected, as individual GO term categories do not refer to genes which form known pathways or complexes but rather represent genes which impact on a broader common biological or molecular process, and each gene might do so in an independent manner. We also looked more broadly at the entire set of genes deregulated by GABPA depletion and, strikingly, amongst genes positively regulated by GABPA, several subnetworks can be identified, one of which relates to known GABPA functions in controlling the cell cycle [9], [14]. Additional subnetworks point to a role for GABPA in controlling different aspects of gene expression and also cytoskeletal activities (Figs. S4). In contrast, fewer subnetworks were detectable amongst the genes negatively regulated by GABPA, with the most prominent one being associated mainly with transcriptional regulation (Figs. S5). To concentrate on the role of GABPA as a direct regulator of genes associated with the cytoskeleton, cell migration and adhesion, we further probed the interconnectivities amongst target genes that are bound and regulated by GABPA and that are annotated with the relevant GO terms. We found that the majority of these genes also formed an interconnected network (Fig. 3A). Four genes from this network, *RAC2*, *RHOF*, *RACGAP1* and *KIF20A*, were taken for further analysis due to their multiple interactions, and likely functional importance as nodes within the network.

First we validated the microarray data for these targets by performing quantitative RT-PCR on MCF10A cells depleted of GABPA (Fig. 3B). Three of the four selected genes (*RAC2*, *RACGAP1* and *KIF20A*) exhibited significant reductions in expression upon depletion of GABPA, while no statistically significant changes were seen on two control genes or *RHOF*, suggesting that the latter is probably a false positive. Similarly, we were able to detect specific binding of GABPA to the regulatory regions of *RAC2*, *RACGAP1* and *KIF20A* in MCF10A cells but no binding to the *RHOF* regulatory region could be detected, reaffirming this as a likely false positive. Importantly, these results confirmed that *RAC2*, *RACGAP1* and *KIF20A* are direct targets for GABPA in MCF10A cells as predicted from ChIP-seq data. Gene expression data showed that at least two of these genes, *RAC2* and *RACGAP1* are not regulated by ELK1, whereas *RHOF* and *KIF20A* require ELK1 for maximal activity (Fig. 2D). Previous ChIP-seq studies did not identify ELK1 occupancy at any of these genes [7] but we wished to confirm this by ChIP-qPCR. All of these genes exhibit detectable ELK1 binding to their regulatory regions (Fig. 2D). However, the binding was relatively low compared to the established ELK1 targets, *CDKL3* and *RFC4* (Fig. 3D). It is not clear whether this level of binding is sufficient to allow ELK1-mediated gene regulation, as observed at *RHOF* and *KIF20A*, but this low level binding apparently has little effect on *RAC2* and *RACGAP1* expression and the latter two genes appear to be specific directly regulated GABPA targets.

These experiments therefore identify *RAC2*, *RACGAP1* and *KIF20A* as likely important nodes in networks associated with the cytoskeleton and cell migration, and these have been verified as direct targets for GABPA-mediated transcriptional activation.

### Key GABPA target genes are involved in cell migration control

**Figure 4:**
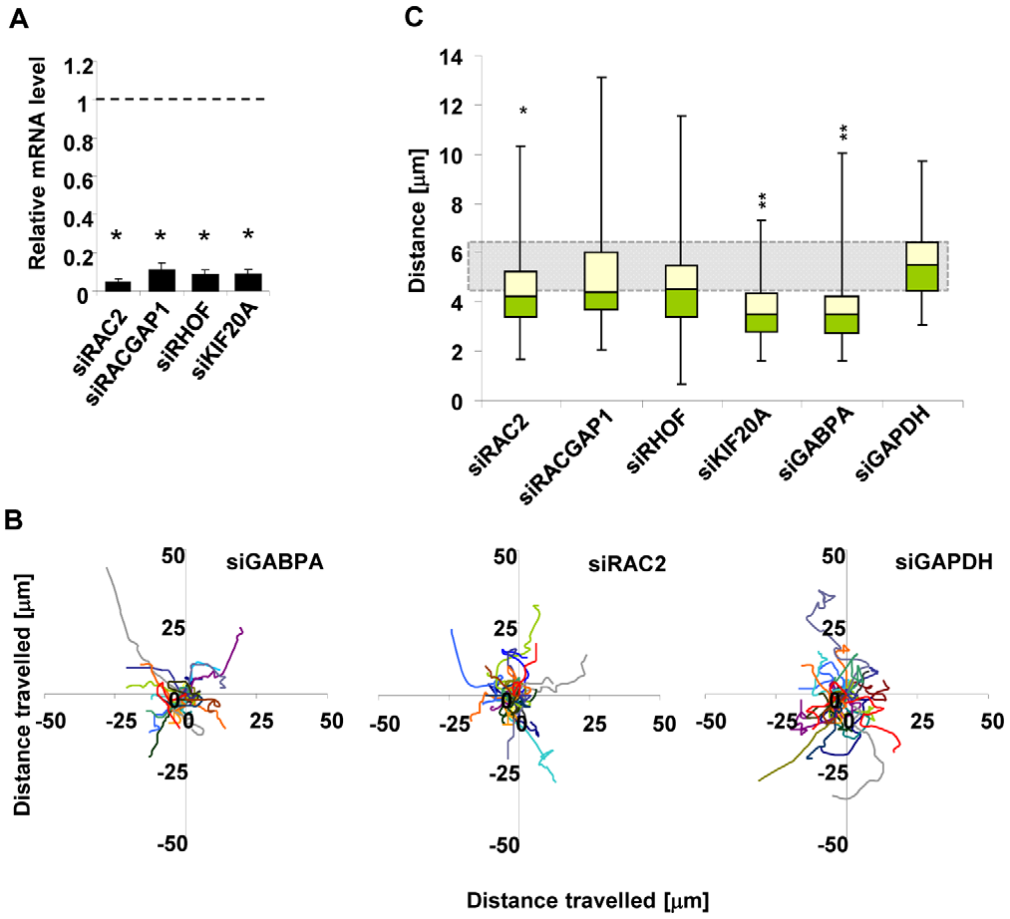
Depletion of direct target genes of GABPA slows down MCF10A cell migration. (**A**) Graph shows the mRNA levels of four GABPA target genes in cells transfected with the respective siRNA species. Values were normalised to control (siGAPDH transfection) and are presented on one chart for clarity. Bars represent average values from three biological repeats with standard deviation. Statistical significance was determined in Student's paired t-tests (*P<0.001). (**B and C**) MCF10A cells were transfected with the indicated siRNAs, starved for EGF for 48 hours, stimulated with media containing 20 ng/ml EGF and imaged for 24 hours. (**B**) Shown are trajectories travelled by cells in the first six hours of live imaging experiments in the presence of the siRNA species indicated above each graph (only three out of the six sets of trajectories are depicted). (**C**) Box plots show the distributions of lengths of trajectories travelled by MCF10A cells transfected with the indicated siRNA species between t = 1 h and t = 7 h after the addition of EGF (which corresponds to t = 0 to t = 6 h of imaging). Data was obtained in three biological repeats of the experiment, in each case ten cells were manually tracked. The green and pale yellow areas correspond to the second and third quartile of the distribution, respectively. The shaded area represents the distribution of distances covered in control siGAPDH-transfected cells. P-values were obtained in a Smirnov-Kolomogorov test (*P<0.05 ** P<0.001).

Our data suggest that GABPA affects cell migration by controlling the expression of a programme of genes associated with this process through both direct and indirect mechanisms. To probe whether the target genes directly activated by GABPA are important for MCF10A cell migration, we investigated whether four of this category of genes *RAC2*, *RHOF*, *RACGAP1* and *KIF20A* play a part in this process. Each of these genes was individually depleted in MCF10A cells by siRNA treatment (Fig. 4A) and the effect on cell migration monitored by single cell tracking. The depletion of *RAC2* had a similar effect to depletion of *GABPA* and caused a significant reduction in the distribution of cells that showed strong migratory properties (Fig. 4B). Similarly, the depletion of *KIF20A* caused a significant reduction in cell migration but neither *RACGAP1* nor *RHOF* depletion affected migration (Fig. 4C). The lack of effect of *RHOF* depletion is predicted based on its identification as a false-positive for GABPA-mediated regulation.

These data therefore provide strong support for a role for GABPA in directly controlling the expression of a group of genes that have key functions in controlling cell migration.

## Discussion

The ETS-domain transcription factors are an excellent model to study how individual members of transcription factor families can elicit specific biological effects. Several genome-wide ChIP analyses have shown that different members of this family show broad overlaps in the genomic regions to which they bind [4]–[7], [15]. However, despite these overlaps, there are groups of binding regions that appear to be uniquely bound by one or a limited subset of family members, and it is thought that it is through these regions that the specific regulatory activities of individual family members are elicited. Indeed, we recently showed that in breast epithelial MCF10A cells, the ETS protein ELK1 binds in a ‘unique’ manner to a set of binding regions, and through these sites, it regulates the expression of a set of genes that are ultimately involved in controlling cell migration [7]. There is another set of ELK1 binding regions that, in another cell type, can also be occupied by a different ETS protein, GABPA, and these regions are not generally associated with genes involved in the migratory properties of these cells. Thus, it was assumed that GABPA would not control cell migration in MCF10A cells but instead would drive different biological processes. Here, we demonstrate that although GABPA likely affects many different biological processes, contrary to expectations, it also plays an important role in controlling cell migration. However, GABPA and ELK1 control cell migration through directly regulating the expression of different profiles of target genes. Thus in this case, the binding regions and hence target gene networks for the ETS proteins are distinct, and yet they ultimately converge to control the same biological process.

Previous studies on GABPA have hinted at a role in controlling cell migration. For example, it was shown that depletion of GABPA reduced the migratory properties of vascular smooth muscle cells [14]. These effects on migration were attributed to its role in controlling the expression of the kinase KIS, and the subsequent effects on phosphorylation and activity of the cell cycle inhibitor p27. However, here we have shown a wider role of GABPA in controlling the expression of genes directly involved in controlling cell migration. In the same study, depletion of GABPA in MEFs reduced the numbers of cells entering the cell cycle [14], which is consistent with previous work that implicated GABPA as a key controller of cell cycle progression [9]. We also find that in MCF10A cells, GABPA plays an important role in controlling the activity of a programme of genes involved in cell cycle control (Fig. 2B; Figs. S3. S4) and it appears to do this by both indirect and direct mechanisms. In keeping with this finding, depletion of GABPA in MCF10A cells leads to changes in their overall cell cycle distributions (data not shown). In another study, the analysis of the entire GABPA regulome led to the identification of many of the functional categories that also appear in our data as potentially directly regulated by GABPA such as “transcriptional regulators” in addition to “cell cycle regulation” [8]. However, by further subpartitioning GABPA targets according to regulatory mode, our study provides further insight and suggests that many of these categories are upregulated by GABPA activity. Indeed, overall the predominant mode of action for GABPA appears to be as a transcriptional activator (Fig. 2A [8]). Conversely, we show that GABPA depletion also causes upregulation of gene expression, implying a repressive role, even in the context of direct target genes. Interestingly, several genes encoding transcriptional repressors (e.g. *NCOR2*, *HDAC5*, *BCL6*, *BCOR*) are upregulated upon GABPA depletion which might then cause some of the observed decreases in gene expression.

In this study we made use of available ChIP-seq data for GABPA to distinguish between likely directly and indirectly regulated targets. While enrichment of GO term categories relating to the cytoskeleton were identified as controlled by GABPA in the entire regulome, these categories were not apparent when direct GABPA targets were analysed, suggesting that the effect of depletion of this factor on cell migration is at least partially secondary. However, importantly, we also uncovered a set of potential key regulators of cell migration that are direct targets for GABPA. It is possible that the number of direct targets is either under or over-estimated due to using ChIP-seq data from a different cell line to MCF10A where the expression studies were conducted. Indeed, *RHOF* appears to be incorrectly designated as a direct GABPA target (Fig. 3). Nevertheless, several of these direct targets were validated in breast epithelial MCF10A cells, and *RAC2* and *KIF20A* were subsequently shown to be important in controlling cell migration in this cell type (Fig. 4). RAC2 is a Rho GTPase that has previously been shown to control the chemotaxis of neutrophils through its effects on the actin cytoskeleton [16]. KIF20A is a kinesin involved in trafficking and has previously been shown to play an important role in late cell cycle progression [17], [18]; thus its effects on migration are a novel finding. However, it is not currently clear whether the effects we see for KIF20A on migration are independent of this activity or are indirectly linked to cell cycle defects caused by its loss. Interestingly, like KIF20A, RACGAP1 has also been implicated in controlling cytokinesis [19] but we see no effect of RACGAP1 depletion on cell migration (Fig. 4). Thus, these two events need not necessarily be linked. While we have analysed a limited number of GABPA target genes here, the final phenotype likely results from changes in the expression of multiple genes controlling cell migration. Indeed, this is the mechanism through which ELK1 affects this process [7], and this type of regulation is more akin to how many microRNAs function, in dampening down the activity of entire pathways rather than acting through a single key regulator (reviewed in [20]). Overall, therefore, GABPA plays a complex role in controlling cell migration through directly affecting the expression of genes encoding key proteins involved in this process, and also by working in a more indirect manner to impact on cell migration.

## Materials and Methods

### Cell culture and imaging, migration assays, RNA interference and RT-PCR

MCF10A cells were grown and all assays were performed as described in [7]. All siRNA duplexes were ON-TARGETplus SMARTpools (Dharmacon) except for GABPA, where a SantaCruz reagent (sc-37100) was also used. Primer pairs used in RT-PCR reactions are listed in Table S2.

### Expression microarray analysis

Expression array experiments were performed in triplicate and analysed as described previously [7] with the following modifications: only MCF10A cells grown in the absence of EGF for 48 hours were used, and filtering of probes with signal lower than background was not applied. One repeat was performed with an ON-TARGET SMARTpool siGABPA and two were performed with the SantaCruz duplex. Data are shown in Table S1 and are deposited with ArrayExpress (E-MEXP-3682).

### Chromatin immunoprecipitation

ChIP experiments using antibodies against ELK1 (Epitomics), GABPA (SantaCruz, sc-22810) and normal rabbit IgG (Millipore) were carried out as described previously [7].

### Bioinformatic and statistical analysis

All overlaps of lists of gene names were performed using an online tool available at http://jura.wi.mit.edu/bioc/tools/compare.php. Networks of protein-protein interactions were created in STRING [13] using physical interaction, coexpression, database and literature mining as proximity criteria at medium stringency. Clustering of STRING networks was performed using an embedded k-means algorithm, with numbers of expected clusters determined empirically. Z-score analysis and the statistical analysis of qPCR and imaging results were carried out as described previously [7].

## Supporting Information

Figure S1 **The effect of GABPA depletion on MCF10A cell phenotype is specific.** (**A and B**) Wound healing assays were performed as in Figure 1C and D, with the use of an alternative siRNA duplex. Instead of time-lapse imaging, cells were fixed 15 hours after EGF stimulation and stained with crystal violet. Shown are representative images of wounds (**A**) and quantification of three biological repeats of the experiment (average values with standard deviations) (**B**).
(TIF)

Figure S2 **Overlaps between GABPA regulated genes and direct ELK1 targets.** Table shows numbers of genes exhibiting a change of expression upon depletion of GABPA (**B**); the overlap of these groups of genes with lists of genes assigned to ELK1 only (**C**) or to both ELK1 and GABPA ChIP-seq regions (**D**); and the overlap of genes up- or down-regulated upon siGABPA transfection and assigned to regions bound by both factors with lists of genes exhibiting a change of expression in cells transfected with siELK1 (**E** and **F**). N/S – no significant bias in distributions between up- and down-regulated genes (Fisher's Exact test).
(TIF)

Figure S3 **Depletion of GABPA causes a profound effect on the expression of genes coding for a network of cytoskeleton- migration- and adhesion-related proteins.** Image shows a STRING-derived network of all genes which exhibit a statistically significant change of expression in MCF10A cells depleted of GABPA and which belong to GO terms associated with the cytoskeleton, cell migration or adhesion as determined by DAVID analysis.
(TIF)

Figure S4 **GABPA directly activates the expression of several functional classes of genes.** Image shows a STRING-derived network of proteins encoded by all genes which exhibit a statistically significant downregulation of expression in MCF10A cells depleted of GABPA and which are associated with GABPA binding DNA regions. The network was clustered using the k-means algorithm provided by the STRING portal, with the number of clusters pre-set to 7 (empirically estimated as optimal). The functions of the proteins within circled clusters were determined through literature- and database mining.
(TIF)

Figure S5 **Genes negatively regulated by GABPA form several small clusters and code for stress-associated proteins.** Image shows a STRING-derived network of proteins encoded by all genes which exhibit a statistically significant upregulation of expression in MCF10A cells depleted of GABPA and which are associated with GABPA binding DNA regions. The network was clustered using the k-means algorithm provided by the STRING portal, with the number of clusters pre-set to 4 (empirically estimated as optimal). The functions of the proteins within circled clusters were determined through literature- and database mining. Several subnetworks of proteins which are not discovered by STRING as clusters share partial functional associations.
(TIF)

Table S1 **Lists of GABPA regulated genes.** Summary of expression microarray data of gene expression changes in MCF10A cells following GAPBA depletion. Direct targets are inferred by comparing to GABPA occupancy as inferred from ChIP-seq analysis (see text for details).
(XLS)

Table S2 **Oligonucleotides used for ChIP- and RT-qPCR.** List of all oligonucleotides used in this study.
(DOCX)
