# Supplementary figures and images for: The ETS Transcription Factors ELK1 and GABPA Regulate Different Gene Networks to Control MCF10A Breast Epithelial Cell Migration
Source: PLoS One. 2012 Dec 20;7(12):e49892. doi: 10.1371/journal.pone.0049892 (PMC3527487; doi:10.1371/journal.pone.0049892)

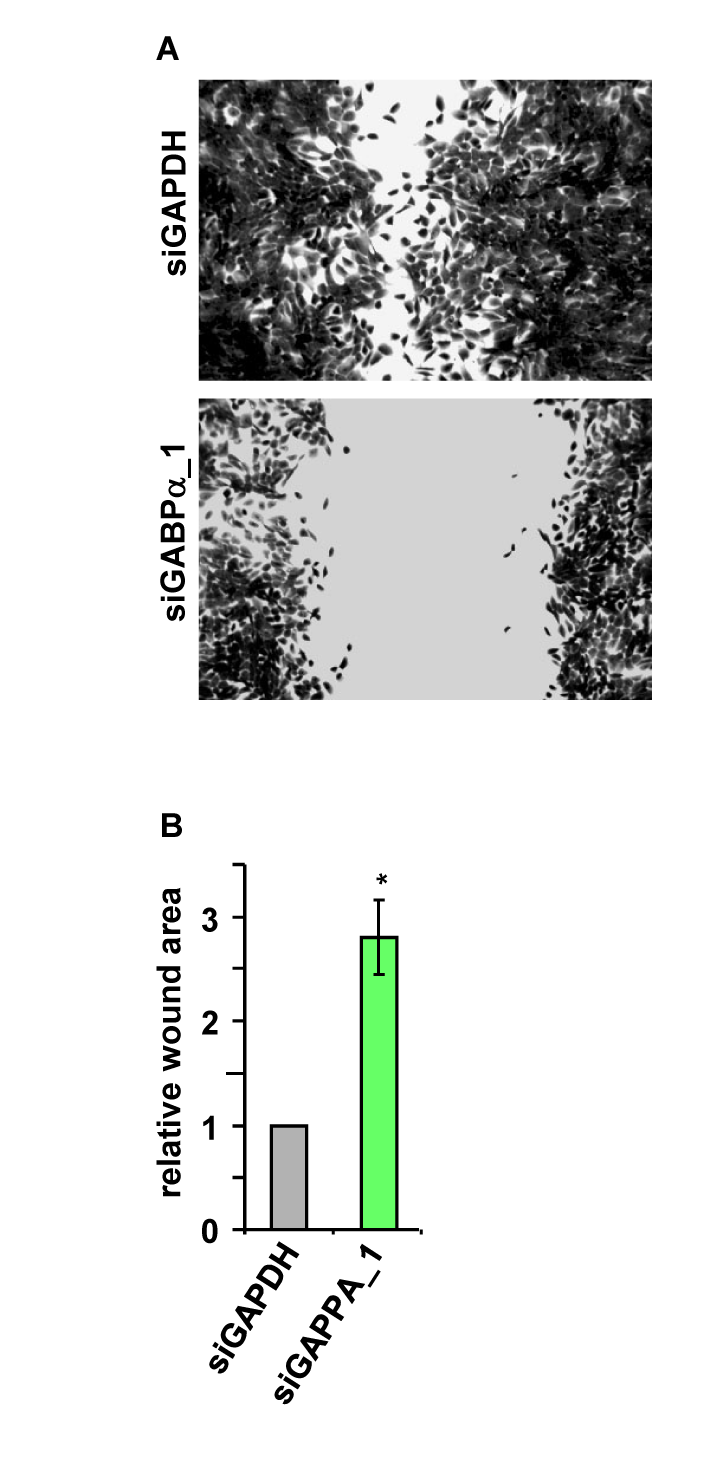

Supplement: Figure S1 — The effect of GABPA depletion on MCF10A cell phenotype is specific. (A and B) Wound healing assays were performed as in Figure 1C and D, with the use of an alternative siRNA duplex. Instead of time-lapse imaging, cells were fixed 15 hours after EGF stimulation and stained with crystal violet. Shown are representative images of wounds (A) and quantification of three biological repeats of the experiment (average values with standard deviations) (B). (TIF) [file pone.0049892.s001.tif]

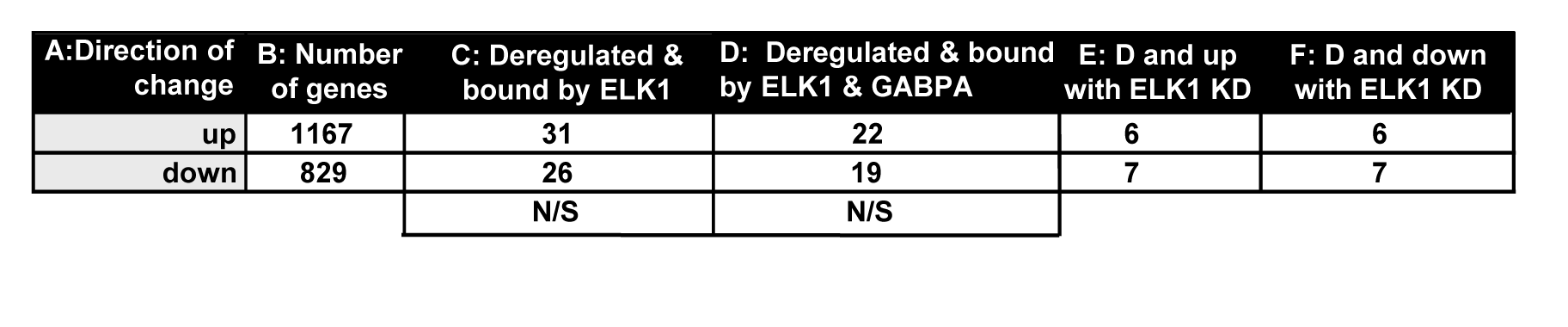

Supplement: Figure S2 — Overlaps between GABPA regulated genes and direct ELK1 targets. Table shows numbers of genes exhibiting a change of expression upon depletion of GABPA (B); the overlap of these groups of genes with lists of genes assigned to ELK1 only (C) or to both ELK1 and GABPA ChIP-seq regions (D); and the overlap of genes up- or down-regulated upon siGABPA transfection and assigned to regions bound by both factors with lists of genes exhibiting a change of expression in cells transfected with siELK1 (E and F). N/S – no significant bias in distributions between up- and down-regulated genes (Fisher's Exact test). (TIF) [file pone.0049892.s002.tif]

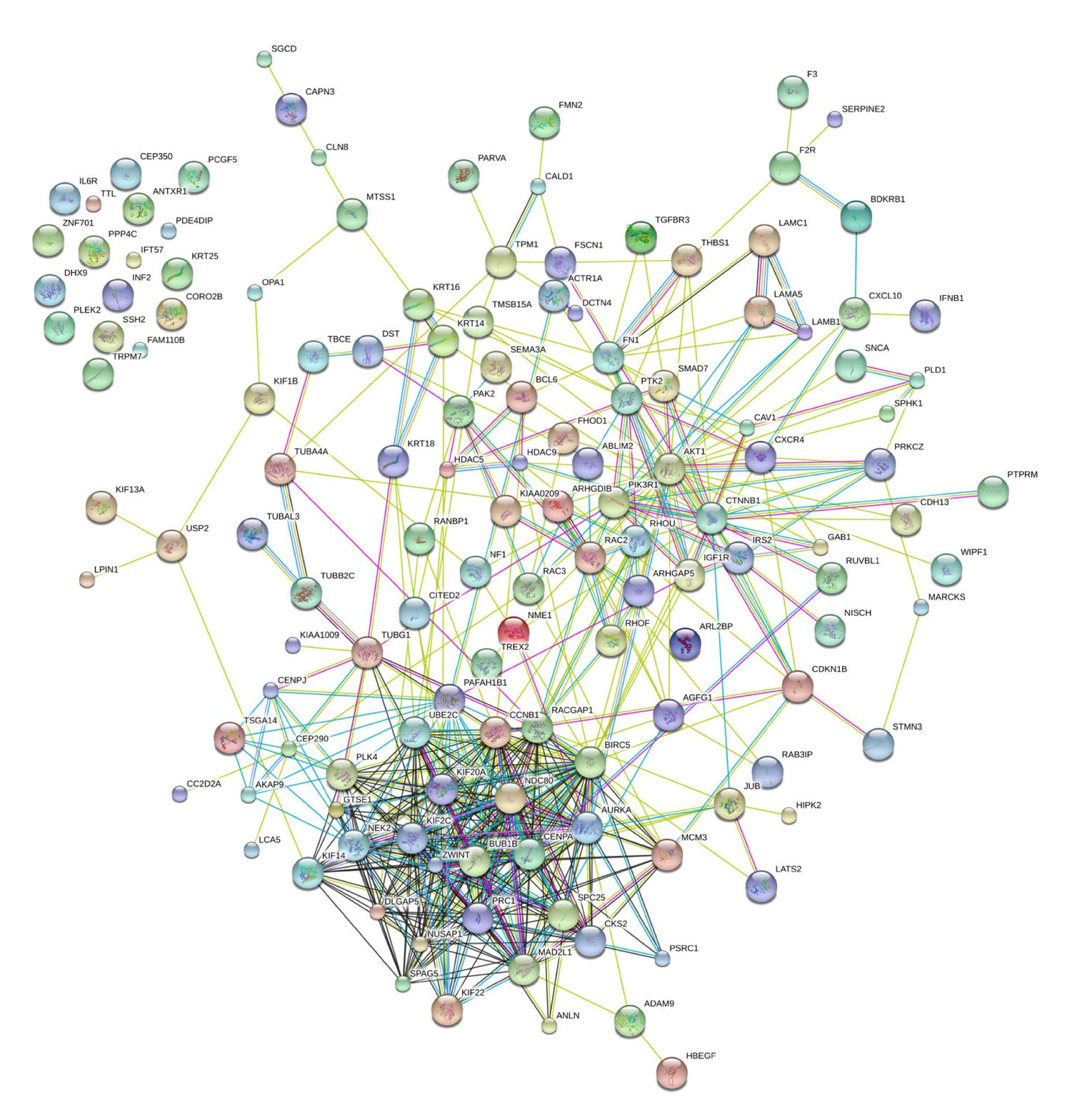

Supplement: Figure S3 — Depletion of GABPA causes a profound effect on the expression of genes coding for a network of cytoskeleton- migration- and adhesion-related proteins. Image shows a STRING-derived network of all genes which exhibit a statistically significant change of expression in MCF10A cells depleted of GABPA and which belong to GO terms associated with the cytoskeleton, cell migration or adhesion as determined by DAVID analysis. (TIF) [file pone.0049892.s003.tif]

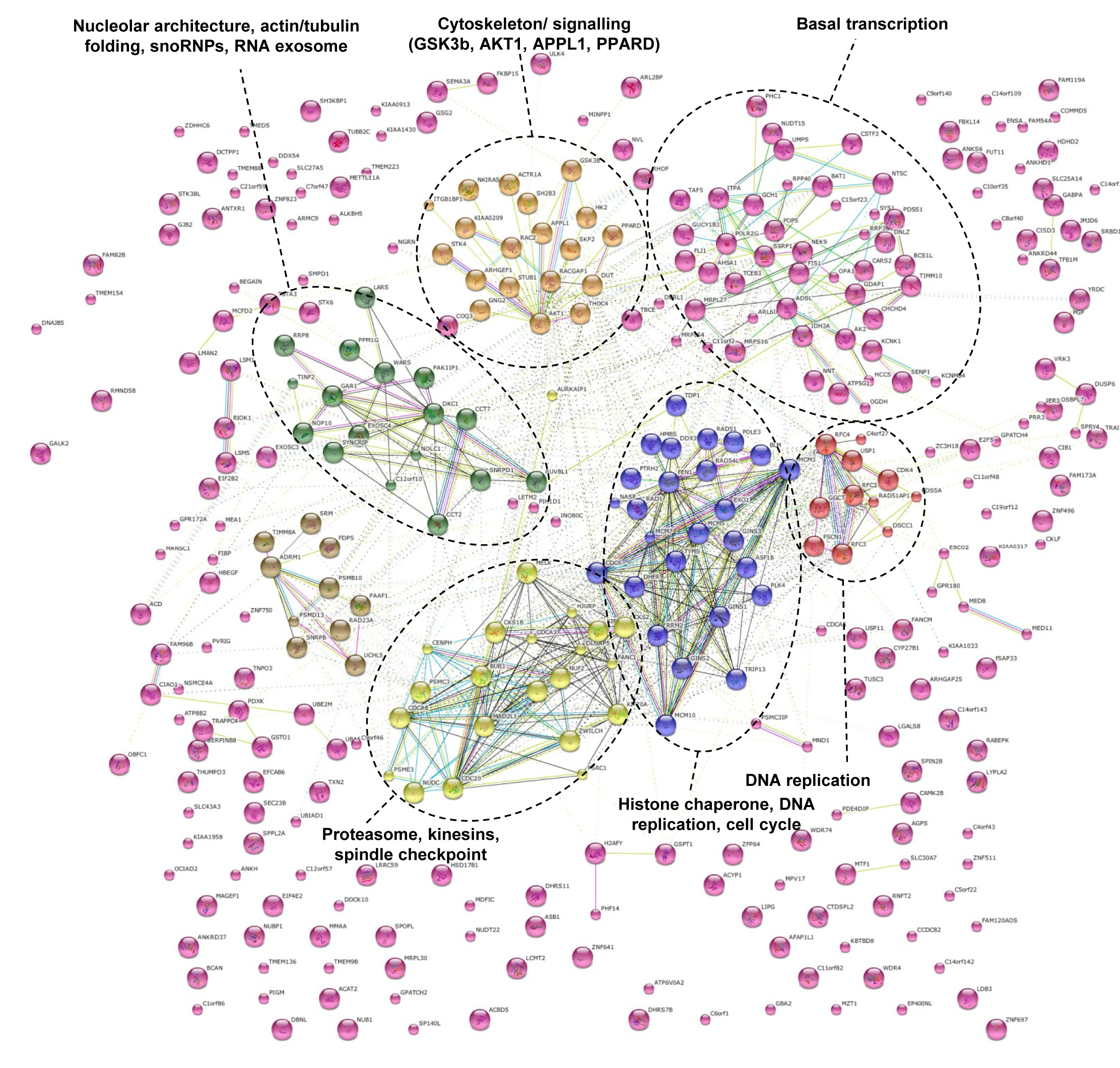

Supplement: Figure S4 — GABPA directly activates the expression of several functional classes of genes. Image shows a STRING-derived network of proteins encoded by all genes which exhibit a statistically significant downregulation of expression in MCF10A cells depleted of GABPA and which are associated with GABPA binding DNA regions. The network was clustered using the k-means algorithm provided by the STRING portal, with the number of clusters pre-set to 7 (empirically estimated as optimal). The functions of the proteins within circled clusters were determined through literature- and database mining. (TIF) [file pone.0049892.s004.tif]

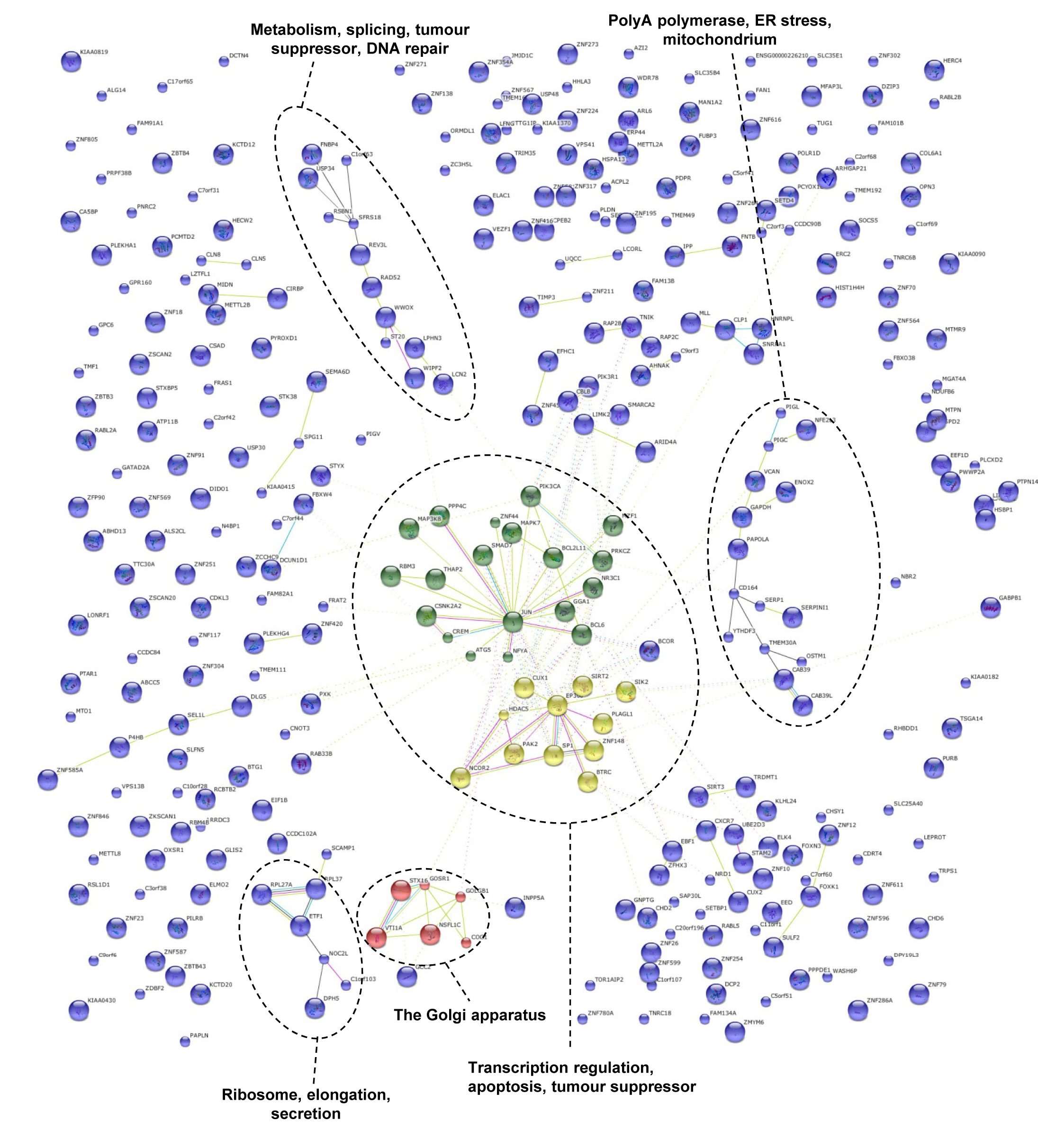

Supplement: Figure S5 — Genes negatively regulated by GABPA form several small clusters and code for stress-associated proteins. Image shows a STRING-derived network of proteins encoded by all genes which exhibit a statistically significant upregulation of expression in MCF10A cells depleted of GABPA and which are associated with GABPA binding DNA regions. The network was clustered using the k-means algorithm provided by the STRING portal, with the number of clusters pre-set to 4 (empirically estimated as optimal). The functions of the proteins within circled clusters were determined through literature- and database mining. Several subnetworks of proteins which are not discovered by STRING as clusters share partial functional associations. (TIF) [file pone.0049892.s005.tif]
