# Supplementary material for: The ETS Transcription Factors ELK1 and GABPA Regulate Different Gene Networks to Control MCF10A Breast Epithelial Cell Migration
Source: PLoS One. 2012 Dec 20;7(12):e49892. doi: 10.1371/journal.pone.0049892 (PMC3527487; doi:10.1371/journal.pone.0049892)
Supplement: Table S2 — Oligonucleotides used for ChIP- and RT-qPCR. List of all oligonucleotides used in this study. (DOCX) [file pone.0049892.s007.docx]

**Oligonucleotides used for ChIP-qPCR**

| ASSOCIATED GENE | PRIMER NUMBER | SEQUENCE | REGION AMPLIFIED (Mar. 2006 (hg18) assembly) | PCR PRODUCT SIZE (bp) |
| --- | --- | --- | --- | --- |
| RAC2 | ADS3477  ADS3478 | GTGTGGAGGCTGGTGAGG  TCTTGGAGAACAGCCTCTGG | chr22:35970204+35970484 | 281 |
| RACGAP1 | ADS3479  ADS3480 | CAAAGGTGGCCATTTTGACT  TAGTCCCGCAGACAGTGAGA | chr12:48705672+48705829 | 158 |
| RHOF | ADS3481  ADS3482 | TTCCTGCCTCAGAGGATGTC  GTGGCTCTCAGAGGCTGAAT | chr12:120712286+120712527 | 242 |
| KIF20A | ADS3483  ADS3484 | TAAGTGCGAATCCACGACAG  GAAGCCTGCAGCTACCAATC | chr5:137542496+137542624 | 129 |

**Oligonucleotides used for RT-qPCR**

| **GENE** | **PRIMER NUMBER** | **STRAND** | **SEQUENCE** |
| --- | --- | --- | --- |
| RAC2 | ADS3485  ADS3486 | FORWARD  REVERSE | GTCTTCCTC ATCTGCTTCTC  TATTTCACCGAGTCA ATCTCC |
| RACGAP1 | ADS3503  ADS3504 | FORWARD  REVERSE | TTTGTGTCCCAG ACTTCTCC  CTGTGATTTCTGCTGCTTCC |
| RHOF | ADS3487  ADS3488 | FORWARD  REVERSE | TCATCAAGTGGTTCCCTGAG  AAG ACGTCCTCCACATTCTC |
| KIF20A | ADS3489  ADS3490 | FORWARD  REVERSE | AGGAAGATCAGGGTTGTGTC  TGACTCCATATGTATAGATGAGCC |
| FLNA | ADS3495  ADS3496 | FORWARD  REVERSE | AGTCTCAGTCAAGTTCAACGA  GCACAGCATACTTATCTTGGTC |
| ABHD2 | ADS3491  ADS3492 | FORWARD  REVERSE | CTACAACTTCCTCATGGCTG  GAACATAAATCCTGTGCAGGT |

**Supplementary Table S2: Oligonucleotides used for ChIP- and RT-qPCR**
